# Reporting of health equity considerations in cluster and individually randomized trials

**DOI:** 10.1186/s13063-020-4223-5

**Published:** 2020-04-03

**Authors:** Jennifer Petkovic, Janet Jull, Manosila Yoganathan, Omar Dewidar, Sarah Baird, Jeremy M. Grimshaw, Kjell Arne Johansson, Elizabeth Kristjansson, Jessie McGowan, David Moher, Mark Petticrew, Bjarne Robberstad, Beverley Shea, Peter Tugwell, Jimmy Volmink, George A. Wells, Margaret Whitehead, Luis Gabriel Cuervo, Howard White, Monica Taljaard, Vivian Welch

**Affiliations:** 1 Bruyere Research Institute, University of Ottawa, Ottawa, ON Canada; 2 School of Rehabilitation Therapy, Queen’s University, Kingston, ON Canada; 3 Infectious Diseases and Prevention Control Branch, Public Health Agency of Canada, Ottawa, ON Canada; 4 Department of Global Health, Milken Institute School of Public Health, George Washington University, Washington, DC, USA; 5 Clinical Epidemiology Program, Ottawa Hospital Research Institute, Ottawa, ON Canada; 6 Department of Medicine, University of Ottawa, Ottawa, ON Canada; 7 Bergen Centre for Ethics and Priority Setting (BCEPS) Department of Global Public Health and Primary Care, University of Bergen, Bergen, Norway; 8 School of Psychology, Faculty of Social Sciences, University of Ottawa, Ottawa, ON Canada; 9 School of Epidemiology and Public Health, Faculty of Medicine, University of Ottawa, Ottawa, ON Canada; 10 Ottawa Methods Centre, Ottawa Hospital Research Institute, Ottawa, ON Canada; 11 Department of Social and Environmental Health Research, London School of Hygiene and Tropical Medicine, London, UK; 12 Section for Ethics and Health Economics, Department of Global Public Health and Primary Care, University of Bergen, Bergen, Norway; 13 Department of Medicine, Faculty of Medicine, University of Ottawa, Ottawa, ON Canada; 14 WHO Collaborating Centre for Knowledge Translation and Health Technology Assessment in Health Equity, Bruyère Research Institute, Ottawa, ON Canada; 15 Faculty of Medicine and Health Sciences, Stellenbosch University, Cape Town, South Africa; 16 Cardiovascular Research Methods Centre, University of Ottawa Heart Institute, Ottawa, ON Canada; 17 Public Health, University of Liverpool, Livepool, UK; 18 Department of Health Systems and Services, Pan American Health Organization, Washington, DC, USA; 19 Campbell Collaboration, Delhi, India; 20 Clinical Epidemiology Program, Ottawa Hospital Research Institute (OHRI), The Ottawa Hospital, Civic Campus, 1053 Carling Avenue, Ottawa, ON K1Y 4E9 Canada

## Abstract

**Background:**

The randomized controlled trial (RCT) is considered the gold standard study design to inform decisions about the effectiveness of interventions. However, a common limitation is inadequate reporting of the applicability of the intervention and trial results for people who are “socially disadvantaged” and this can affect policy-makers’ decisions. We previously developed a framework for identifying health-equity-relevant trials, along with a reporting guideline for transparent reporting. In this study, we provide a descriptive assessment of health-equity considerations in 200 randomly sampled equity-relevant trials.

**Methods:**

We developed a search strategy to identify health-equity-relevant trials published between 2013 and 2015. We randomly sorted the 4316 records identified by the search and screened studies until 100 individually randomized (RCTs) and 100 cluster randomized controlled trials (CRTs) were identified. We developed and pilot-tested a data extraction form based on our initial work, to inform the development of our reporting guideline for equity-relevant randomized trials.

**Results:**

In total, 39 trials (20%) were conducted in a low- and middle-income country and 157 trials (79%) in a high-income country focused on socially disadvantaged populations (78% CRTs, 79% RCTs). Seventy-four trials (37%) reported a subgroup analysis across a population characteristic associated with disadvantage (25% CRT, 49% RCTs), with 19% of included studies reporting subgroup analyses across sex, 9% across race/ethnicity/culture, and 4% across socioeconomic status. No subgroup analyses were reported for place of residence, occupation, religion, education, or social capital. One hundred and forty-one trials (71%) discussed the applicability of their results to one or more socially disadvantaged populations (68% of CRT, 73% of RCT).

**Discussion:**

In this set of trials, selected for their relevance to health equity, data that were disaggregated for socially disadvantaged populations were rarely reported. We found that even when the data are available, opportunities to analyze health-equity considerations are frequently missed. The recently published equity extension of the Consolidated Reporting Standards for Randomized Trials (CONSORT-Equity) may help improve delineation of hypotheses related to socially disadvantaged populations, and transparency and completeness of reporting of health-equity considerations in RCTs. This study can serve as a baseline assessment of the reporting of equity considerations.

## Summary points

*What is already known on the topic*

- The Consolidated Reporting Standards for Randomized Trials (CONSORT) Statement provides a list of items that are required to be reported for all randomized trials
- The CONSORT-Equity 2017 provides an extension to the CONSORT Statement for transparent reporting of health-equity-relevant randomized trials

*What this study adds*

- Pilot of CONSORT-Equity items on a random sample of 200 individually and clustered equity-relevant randomized trials, suggests that one third of the trials reported subgroup analysis related to social determinants of health

## Background

Policy-makers in most countries have committed to reducing inequalities in the Sustainable Development Goals [1]. There is a great need for more empirical evidence on efforts to redress health inequities. Health inequity is defined as *“differences in health that are unnecessary, avoidable, unfair, and unjust”* [2]. Fairness is a normative concept, and there are differing perspectives on what is considered unfair and these may differ across settings and time [3]. Health inequalities are considered unfair when they can be avoided, prevented, or mitigated [4]. Not all health differences are health inequities; for example, age differences in Alzheimer’s disease prevalence would not be considered an age-related inequity because the risk increases with increasing age [5]. Also, judgments about fairness may need to take into account opportunity costs in sectors outside of health when redressing health inequalities.

Throughout this paper, we use the term “socially disadvantaged” to denote that people are disadvantaged by differences in distribution of power and resources which structure their living and working conditions and affect their opportunities for health [4]. We recognize that this terminology may be seen as labeling or stigmatizing, which is not our intent. Some people and populations may prefer other terms to fit their context such as marginalized, living in a vulnerable situation or under-served [6].

Different frameworks are available to describe factors associated with health inequity. We use the mnemonic PROGRESS-Plus which stands for place of residence, race/ethnicity/culture/language, Occupation, gender/sex, religion, education, socioeconomic status, and social capital [7, 8]. We also recognize additional characteristics as the “Plus” characteristics, such as: individual characteristics (e.g., age, disability); features of relationships; and time-dependent transitions (i.e., when a person is temporarily at a health disadvantage) [8–10]. As stated above, differences in health outcomes across these characteristics are not always inequitable. We only consider them to be inequities when differences in health across these characteristics are produced or exacerbated by social disadvantage. For example, the difference in average life expectancy across neighborhoods associated with neighborhood-level socioeconomic deprivation in the UK and education levels in Norway is considered inequitable by many [11].

Randomized controlled trials (RCTs) can provide evidence about the effectiveness of an intervention and of the effects on health equity. However, many trials do not include people who may experience social disadvantage, and even if they do, results are rarely disaggregated for specific populations [3]. Under-representation of many populations that commonly experience social disadvantage has been well-documented; people living on low income, ethnic minorities, women, and older adults are often under-enrolled or excluded [12–14] despite ethical guidelines typically stating that under-representation should be avoided [15]. When trials do include adequate numbers of participants representing these groups, the authors often fail to report basic sociodemographic details, such as the socioeconomic status of participants [16], and rarely consider subgroup hypotheses across PROGRESS-Plus characteristics [17, 18]. Carefully planned subgroup analyses can play a vital role in equity-relevant trials. For example, they can be used to investigate whether an observed total treatment effect is consistent across subgroups in the population [19]. They can also be used to identify subgroups with better or worse outcomes, or those experiencing potential harms. Pre-planned subgroup analyses can be used to test intervention effects in groups specifically targeted by an intervention, or believed to be resistant to treatment. However, subgroup analyses are subject to multiple methodological challenges which can complicate their interpretation, including increased risks of type I error due to multiple testing and low statistical power [20]. Some reviews have found that subgroup analyses are often poorly justified, infrequently pre-specified, and inadequately reported [21]. While such challenges may seem to discourage their use, lack of disaggregation or descriptive detail means that judgments about the policy relevance of the evidence to people who live with social disadvantage may be difficult for decision-makers who are using the evidence to inform and implement policies, programs, or individual patient care. Additionally, this lack of disaggregation limits the information available for systematic review authors and makes it impossible to explore differential effectiveness of the intervention. While underpowered subgroup analyses in trials should be interpreted with caution, they can provide useful information about the nature and direction of any potential effect and, when combined with data from other studies, can make more informative meta-analyses [22]. This inadequate consideration of potentially disadvantaged populations is often described as a limitation by those who rely on research to make decisions concerning socially disadvantaged populations [23, 24].

To address these concerns and the calls made in international public health policy documents as well as commitments from research funders and journal editors [25–28], we have developed a reporting guideline for health-equity-relevant RCTs (CONSORT-Equity), which aims to increase the transparency and completeness of reporting of equity analyses to improve their usefulness for health-equity-relevant decision making [29].

Not all published trials are relevant to decisions concerning equity [3]. We define health-equity-relevant trials using the following criteria:

1. Does the study include individuals or populations who experience social disadvantage (across one or more of PROGRESS-Plus characteristics) within the setting and context of the study?
2. If yes, does the study assess the effects of the intervention on the health of people who experience social disadvantage by either:
  - Exclusively focusing on individuals or populations who experience social disadvantage; or
  - Including a heterogeneous group with assessment of the differential impacts of the intervention across one or more PROGRESS characteristics?

Cluster randomized trials (CRTs), that is, trials in which the units of randomization are coherent groups, communities, schools, or medical practices, rather than separate individuals, have unique methodological requirements, and also, unique equity considerations [30]. They pose additional challenges for assessing effects on health equity compared to individually randomized trials because clusters may include individuals experiencing different levels of social disadvantage and/or clusters may have different experience of social disadvantage. In addition, populations that are socially disadvantaged across one or more PROGRESS-Plus characteristics may be hidden within the clusters.

This study aimed to provide a descriptive assessment of the reporting of health-equity considerations in a random sample of 200 health-equity-relevant trials (100 individually randomized trials, RCTs and 100 CRTs). The preliminary results of this study were used to inform the development of the CONSORT-Equity 2017 Reporting Guideline [29]. We also used this as an opportunity to conduct a “baseline” assessment of the reporting of health-equity considerations in these trials; that is, before the publication of the CONSORT-Equity 2017 guidelines, by tabulating the proportion of trials reporting each CONSORT-Equity 2017 item in these equity-relevant trials.

## Methods

### Study protocol

This methods study was conducted as part of a larger project to develop a reporting guideline for health-equity relevant trials for which a protocol was published [31].

### Eligibility criteria

We included a random sample of health-equity-relevant trials (100 RCTs and 100 CRTs) using the criteria listed in our conceptual framework (above) [3].

Individual and cluster randomized trials with primary trial reports were eligible.

### Searching

We developed a search strategy in collaboration with a librarian scientist (JM) for health- equity-relevant trials using both text-words and MeSH headings (Appendix 1). We tested this strategy with a reference set of 12 identified health-equity-relevant trials. The search was modified and retested multiple times to ensure that a sufficient number of health-equity-relevant trials were retrieved. We searched MEDLINE, Sociological abstracts and Econlit to encompass medical, public health, and international development interventions. We focused on these three major databases that were likely to include an adequate population of equity-relevant trials from which we could identify a sample to assess. We conducted the search up to 5 May 2015 and restricted the search to the years 2013–2015 as we were interested in describing the most recent reporting practices.

### Screening

Records were exported to Excel and sorted in random sequence using the built-in random-number generator; the titles and abstracts were then screened until the target sample size for RCTs and CRTs was achieved. Titles and abstracts and the full texts of potentially health-equity-relevant trials were screened independently, by two researchers, using Covidence software [20]. Conflicts were resolved through discussion at weekly team meetings.

At the title/abstract screening phase, decisions were based solely on whether the content of the abstract met the eligibility criteria even though some trials may have reported on health equity in the full text.

### Developing and testing the data extraction form

We developed and pretested a data extraction form using Microsoft Excel (see Appendix 2 for data extraction items). Through pilot-testing, we developed a data dictionary to ensure consistent extraction of all items and revised this for clarification with the team.

We decided to focus on the PROGRESS characteristics for this descriptive study. We also assessed whether the included RCTs and CRTs reported baseline characteristics for the additional PROGRESS- “Plus” characteristics. However, we did not extract details on these “Plus” populations nor on whether subgroup analyses were reported across these “Plus” populations.

### Data extraction and verification

Our extraction form was used to capture data on descriptive characteristics including the purpose of the intervention (whether the trial included only a socially disadvantaged population or whether the trial included general populations in which there may be socially disadvantaged people), and study design and characteristics of the statistical analyses, including the reporting of subgroup analysis. We also collected data on whether there was any adjustment for PROGRESS characteristics as covariates. We assessed whether health-equity considerations were reported, drawing from items from prior work on reporting equity considerations [3, 29, 32, 33]. Details on health-equity considerations were collected from the title/abstract, introduction, methods, eligibility criteria, population characteristics, results, subgroup analysis, interpretation of applicability and discussion. All of these items were pre-planned and defined (Appendix 2).

For subgroup analysis across PROGRESS characteristics, we assessed the quality of reporting using the Yusuf criteria [34]. We chose the Yusuf criteria because they cover the important concepts from other sets of criteria regarding the credibility of subgroup analyses [35, 36] and we considered the criteria feasible to assess. We decided a priori to not assess subgroup analyses across additional PROGRESS-Plus characteristics, such as age or temporary characteristics, since some of these are not amenable to subgroup analyses (such as temporary situations). The Yusuf criteria consist of four questions:

1. Subgroup analysis pretested or planned a priori to the study commencement
2. Hypothesis or rationale for the analysis provided
3. Statistical test for interaction performed between the subgroups
4. Overall treatment results emphasized more than the findings of the subgroup analysis

Data were extracted independently by two data extractors (NA, CC, ETG, MHJ, SL, SN, LSN, WM, JP, KR, CS, SS, HS, MY) working in assigned pairs. The team of data extractors was trained in using the data extraction form and met weekly to discuss any clarity issues with the data extraction items. These pairs met weekly to resolve conflicts and to reach consensus. When necessary, disagreements were discussed with the broader team.

### Data analysis

We used IBM SPSS Statistics (Version 25) to calculate the frequency of reporting each item for all RCTs and CRTs in our sample. We cross-tabulated the data for each item to assess the frequency of reporting for RCTs and CRTs.

## Results

### Search results

**Fig. 1:**
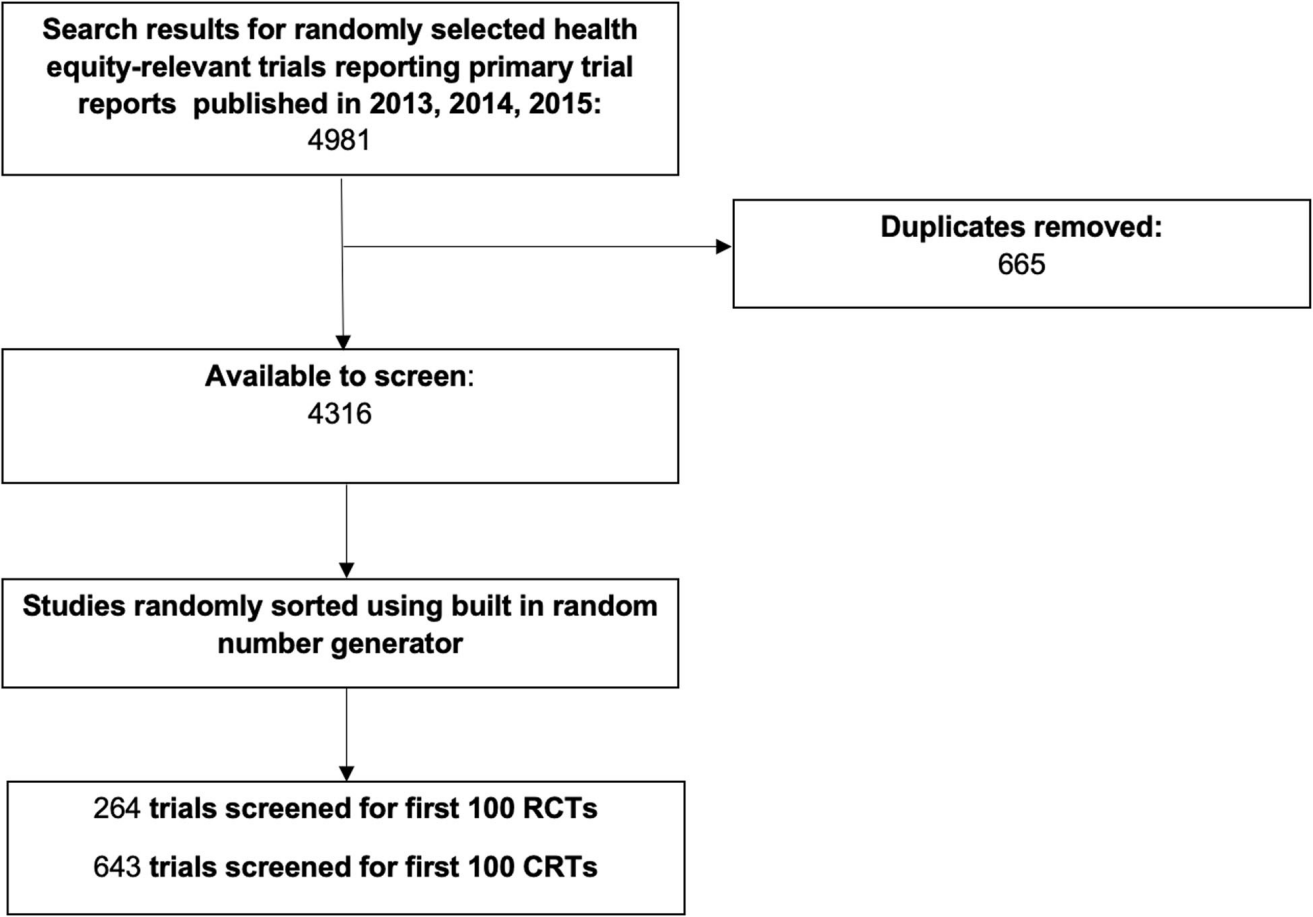
Study flow diagram. *RCT* randomized controlled trial, *CRT* cluster randomized trial

We identified 4981 records from the search. After removing duplicates, we screened 4316 records at the title and abstract stage. We sorted all studies in random sequence and we screened in random sequence until the target sample size was met. We assessed a total of 264 trials to select the first 100 RCTs and 643 trials to select the first 100 CRTs (see Fig. 1).

### Included studies

The characteristics of the included 100 CRTs and 100 RCTs are presented in Table 1. Overall, 60% (119 studies) of the 200 included studies had an explicit health-equity objective (64% CRTs, 55% RCTs). A third of these studies (31%) were conducted in a resource-constrained setting as defined by the trialists (40% CRT, 22% RCTs), of which most were conducted in low- and middle-income countries (LMICs) (20%) (28% CRTs, 11% RCTs). The majority of trials had interventions that focused on socially disadvantaged populations (overall 79%; 78% CRT, 79% RCT).

**Table 1:** Characteristics of included studies

	Cluster RCTs ***N*** = 100	Individually randomized trials ***N*** = 100	Total ***N*** = 200
	(%)	(%)	*N* (%)
Publication year			
2013	51	45	96 (48)
2014	40	49	89 (45)
2015^a^	9	6	15 (8)
Study had an explicit objective pertaining to equity	64	55	119 (60)
Study was reported as conducted in a resource-constrained setting^b^	40	22	62 (31)
Lower- or middle-income country	28	11	39 (20)
Public hospital	1	2	3 (2)
Conflict zone	1	0	1 (0.5)
Other	13	14	27 (14)
Study population^b^			
Students in primary or secondary school	27	11	38 (19)
Workers	3	3	6 (3)
Community members	41	36	77 (39)
Patients	19	46	65 (33)
Members of a particular professional group, such as health professionals or teachers	3	1	4 (2)
Other	–	11	11 (6)
Unit of randomization			
Individuals		100	100 (50)
Schools	27		27 (14)
Workplace	3		3 (2)
Community or community organization	26		26 (13)
Medical practice	12		12 (6)
Other	32		32 (16)
Participants were recruited from:			
Workplace	5	1	6 (3)
School	27	10	37 (19)
Other	68	89	157 (79)
Studies that reported using special or tailored recruitment to increase enrollment of individuals who are members of socially disadvantaged populations	11	32	43 (22)
Type of study			
Focused on socially disadvantaged group	78	79	157 (79)
Universal	18	7	25 (13)
Both focused and universal	4	14	18 (9)

^a^The search was conducted on 5 May 2015

^b^Studies could be classified as fitting more than one type of setting and more than one type of unit of randomization; therefore, numbers do not add up to 100. *RCT* randomized controlled trial

For CRTs, the majority randomized schools (27% of CRTs), communities, or community organizations (26%), medical practices (12%), and workplaces (3%), while the remaining trials (32%) used “other” units of randomization such as families, households, or geographical areas.

### Reporting of health-equity considerations

**Fig. 2:**
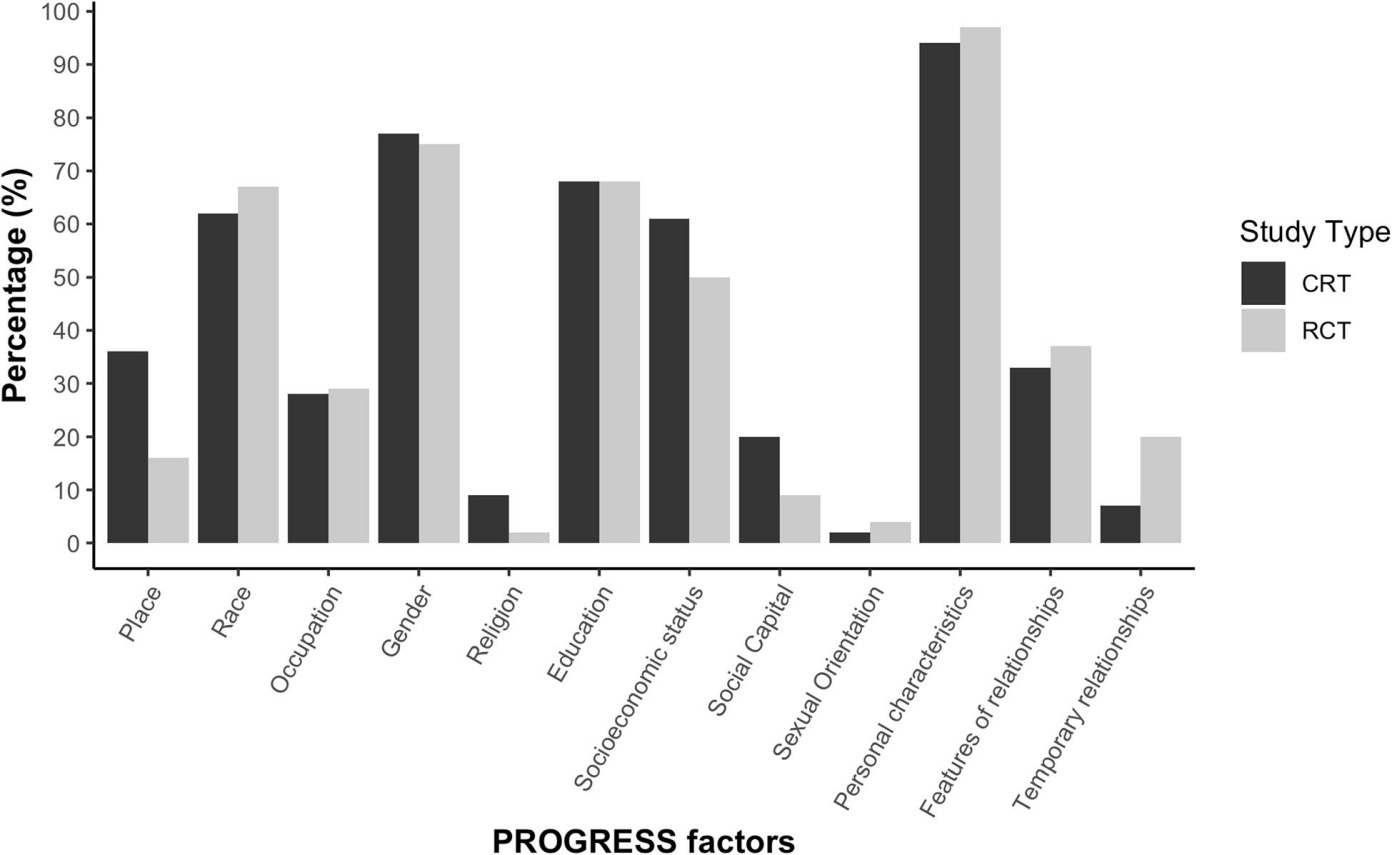
Study baseline descriptive characteristics defined by PROGRESS characteristics

The prevalence of reporting of PROGRESS characteristics was similar between CRTs and RCTs (Fig. 2). Sex was the most commonly reported characteristic (76% of studies) (77% CRTs, 75% RCTs). The least commonly reported characteristic was sexual orientation, which was reported in 3% of studies (2% CRTs, 4% RCT). Additional personal characteristics associated with health inequities were reported by 96% of our sample (94% CRTs, 97% RCTs). Of these additional characteristics, the most commonly reported characteristic was age (41%).

There were few differences in reporting between RCTs and CRTs except that 36% of CRTs reported the place of residence of the participants compared to 16% of RCTs. Social capital, which we assessed as social connections between individuals within a community or household, was reported in 20% of CRTs compared to 9% of RCTs. Social capital was mainly operationalized as marital status. Religion was reported in 9% of CRTs and 2% of RCTs. The higher reporting of place of residence, social capital, and religion in CRTs vs. RCTs may be related to the need to show whether randomization was successful in balancing these characteristics across clusters.

Distribution of PROGRESS-Plus (place of residence, race/ethnicity/culture/language, occupation, gender/sex, religion, education, socioeconomic status, social capital, and additional characteristics) and sexual orientation considerations in 100 CRTs and 100 RCTs.

Overall, 37% of our included studies reported a subgroup analysis across PROGRESS characteristics (25% of CRTs, 49% of RCTs). The most commonly reported subgroup analysis was by sex (13% CRTs, 24% RCTs), followed by race/ethnicity/culture/language (9% overall, 5% CRTs, 13% RCTs). Other PROGRESS subgroup analyses focused on socioeconomic status (4% overall, 5% CRTs, 3% RCTs), and place of residence (3% overall, 3% CRTs, 2% RCTs). Subgroup analyses of other PROGRESS characteristics were reported in less than 5% of studies.

We assessed whether studies reported adjustment for PROGRESS characteristics in their analyses. Adjustment for these characteristics might show that data were available for disaggregated presentation of results or appropriate subgroup analyses; the absence of such results might indicate a missed opportunity for equity-relevant research. Out of all studies, 23% adjusted for gender/sex, 15% for education, 15% for race/ethnicity/culture, 4% for socioeconomic status, and 3% for place of residence. Less than 10% of studies adjusted for the other PROGRESS characteristics in their analyses.

Overall, 71% of the included studies discussed the applicability of their results with regards to one or more PROGRESS characteristics (68 CRTs, 73 RCTs). In addition, 26% of those which discussed applicability (51 out of 200) with regards to a PROGRESS characteristic also reported a subgroup analysis across this characteristic Table 2.

**Table 2:** Reporting of equity considerations in health-equity-relevant randomized trials

	Cluster RCTs ***N*** = 100	Individual RCTs ***N*** = 100	Total ***N*** = 200
	(%)	(%)	*N* (%)
Studies with any subgroup analysis across PROGRESS characteristics	25	49	74 (37)
Primary reported subgroup analysis across PROGRESS characteristics:			
Place of residence	3	2	5 (3)
Race/ethnicity/culture	5	13	18 (9)
Occupation	0	1	1 (0.5)
Gender/sex	13	24	37 (19)
Education	1	3	4 (2)
Socioeconomic status	3	5	8 (4)
Social capital	0	1	1 (0.5)
PROGRESS characteristics adjusted for in analysis:			
Place of residence	7	3	10 (5)
Race/ethnicity/culture	11	18	29 (15)
Occupation	1	5	6 (3)
Gender/sex	17	29	46 (23)
Religion	1	2	3 (2)
Education	14	15	29 (15)
Socioeconomic status	11	14	25 (13)
Social capital	0	2	2 (1)
Applicability/generalizability/external validity discussed across any PROGRESS characteristic	68	73	141 (71)
Studies that had conducted a subgroup analysis and discussed equity with regards to the applicability of the evidence	18	33	51 (26)

For those studies with subgroup analysis, approximately two thirds met the Yusuf criteria for quality of subgroup analysis for pre-planned analysis (68%), rationale provided (61%), statistical test for interaction (73%), and the overall results emphasized more than the subgroup findings (70%) (Table 3). Only one randomized controlled trial (RCT) [37] and no cluster randomized controlled trials (CRTs) mentioned whether these subgroup analyses were informed by a-priori power calculations.

**Table 3:** Yusuf criteria on quality of subgroup analyses (for cluster randomized controlled trials (CRTs) and randomized controlled trials (RCTs) with subgroup analysis)

Yusuf criteria	Cluster RCTs ***N*** = 25	Individual RCTs ***N*** = 49	Total ***N*** = 74
	(%)	(%)	*N* (%)
Subgroup analysis pretested or planned a priori to the study commencement	68	67	50 (68)
Hypothesis or rationale for the analysis provided	60	61	45 (61)
Statistical test for interaction performed between the subgroups	80	70	54 (73)
Overall treatment results emphasized more than the findings of the subgroup analysis	64	73	52 (70)

### Completeness of reporting

Very few of the included studies used the term “equity” in their title (2%). However, other terms were used in the title such as words describing PROGRESS characteristics, such as “low-income.” Sixty-one percent (61%) of included trials reported on health-equity analysis or the extent of the applicability of the results across PROGRESS characteristics in the abstract. Twice as many individually randomized trials analyzed their results using subgroup analysis across one or more PROGRESS characteristics (49% compared to 25% of cluster randomized trials).

Reporting of health-equity PROGRESS characteristics was highest in the background sections of the included studies, with 73% of included studies describing anticipated differences in baseline risk or intervention acceptability, coverage, or effectiveness across population subgroups defined by PROGRESS. We assessed that 60% of these equity-relevant studies reported an explicit objective related to health equity.

Most studies were focused on populations experiencing social disadvantage (79%). Only 25% of studies reported on individual or community engagement processes.

## Discussion

This study was conducted to inform the development of a reporting guideline for health-equity-relevant trials (CONSORT-Equity) [29] and to serve as a baseline assessment of quality of reporting before publication of the CONSORT-Equity extension. The CONSORT Statement and its many extensions provide guidance for reporting trials completely and transparently and the health-equity extension focuses on items that improve the reporting of equity-relevant trials.

In our sample of health-equity-relevant trials, 25% of CRTs and 49% of RCTs reported a subgroup analysis by a PROGRESS characteristic. Of these, the most commonly reported subgroup analysis was across sex and it was only reported in 19% of studies (13% CRT, 24% RCT). Other PROGRESS characteristics were assessed using subgroup analyses in less than 10% of the studies included in our sample. There were almost twice as many studies which adjusted for PROGRESS characteristics as studies which performed subgroup analyses. While the approach to subgroup analysis differs across disciplines, these studies may represent a missed opportunity for understanding effects in socially disadvantaged populations. About two thirds of these subgroup analyses met one or more of the Yusuf criteria for quality of subgroup analyses. Only one out of 200 trials reported formally planning for a subgroup analysis during the design of the trial, by considering the sample size and power available for analysis. Because sample-size calculations are usually based on the primary comparison of interest; that is, between treatment arms, rather than on differential effects within subgroups, it is likely that many studies may have been underpowered for subgroup analyses. We believe that, even with insufficient power, if data were presented as disaggregated by important PROGRESS characteristics in all randomized trials, then it could be used for hypothesis-generation for future studies as well as in subsequent meta-analyses or other studies where greater power could be achieved. However, the results should be interpreted with caution and authors should state that type-II errors are possible.

CRTs had greater prevalence of reporting baseline assessment of place of residence and socioeconomic status than RCTs, possibly because balance across these characteristics is often checked to assess adequacy of cluster randomization. However, CRTs were about half as likely to report subgroup analyses across one or more PROGRESS characteristics than RCTs. Possible reasons are that CRTs have larger sample-size requirements than RCTs and there may have been inadequate numbers of clusters and/or participants to allow subgroup analyses to be conducted, and because statistical analyses are more complicated in CRTs. However, it may also be that we found that CRTs are more likely to be focused on an equity issue (64% compared to 55% of RCTs) and more likely to be conducted in a resource-poor setting (40% compared to 22%) and, therefore, conducting subgroup analyses may have been less relevant.

Our study has some limitations. First, we selected studies which met our criteria for being health-equity-relevant; therefore, the study was not designed to consider how well CONSORT-Equity items are reported in the clinical trials’ literature in general. We did not extract descriptive information about each included study, such as the type of intervention being assessed, so cannot assess whether reporting is different depending on the field of study. We have also only provided a description of what has been reported in these trials and we have not made judgments about how the studies were conducted or what should have been reported. We excluded studies at the abstract stage if they did not meet our criteria for health-equity-relevance which may have excluded some studies that did report on equity analyses in the full text but not the abstract during our screening process. This may have resulted in missing some studies which were health-equity-relevant. It is not possible to determine whether these would have different design or reporting characteristics from those included in our sample. Our search was restricted to the most recent years (2013–2015) as we were interested in describing the most recent reporting practices to inform the development of the CONSORT-Equity reporting guideline. It is important to bear in mind that the reporting of clinical trial results is usually dictated by their study protocols, which typically are 4 to 6 years older than the actual publication. Therefore, we may not have captured the very latest improvements in methods to report health-equity-relevant information from randomized trials. Finally, we did not assess all the CONSORT-Equity items because the final version of the equity extension was developed in 2017 after we had completed data extraction. The items which were not collected were added at the CONSORT-Equity meeting or during the development and included items related to eligibility, context, comparator, stratified randomisation, ethical clearance, implementation, intervention-generated inequities and limitations (e.g., power) to assess effects on health equity.

Our results provide a baseline estimate of the completeness of reporting of the recommended CONSORT-Equity items. These results show that even though these were all health-equity-relevant trials, only 73% described a rationale for their focus on equity socially disadvantaged population, only 25% reported engagement with communities or individuals who are socially disadvantaged, and only 20% mentioned importance of outcomes for socially disadvantaged populations. Because PROGRESS-Plus characteristics may be sensitive, these findings may be limited by the information that participants were willing to disclose [38].

In this sample of health-equity-relevant trials, we found few subgroup analyses that could inform equity decisions. However, many more trials recorded equity-relevant baseline descriptors, which suggests that there was an opportunity to consider how the analyses might inform equity considerations, including a-priori specification of hypothesis tests about treatment effects across relevant subgroups. Our findings concur with other studies which have found little detail about characteristics, such as gender and race/ethnicity/socioeconomic status, in studies to inform decisions about mitigating or redressing health inequities [16, 39–41]. The added value of this paper is that we have identified a dearth of studies reporting adequate consideration of relevant subgroup hypotheses and pre-planned subgroup analyses across several PROGRESS characteristics. This limits their usefulness for informing decisions where health equity is an important consideration. In addition, we have identified deficiencies in reporting details such as the rationale for subgroup analyses and power for these analyses as well as details about the process of recruitment, engagement with people with lived experience and reporting of disaggregated data. This information can be used to guide future research and research reporting.

When health-equity considerations are poorly reported in trials, the applicability of the evidence cannot be fully assessed, and the research will be of less value for reaching important health-policy goals, including Sustainable Development Goals SDG5 (gender equality) and SDG10 (inequality within and between countries) and the calls to provide universal health coverage [42–46]. When these population characteristics are adjusted for but not compared, we are unable to make any conclusions about their potential impact on differential effectiveness of the intervention. This can result in a loss of important policy-relevant or equity-relevant information. In addition, we cannot determine whether populations are included but hidden within trials or have purposefully been excluded or under-represented, in which case the validity of the results to determine prevention and care for the under-represented groups is compromised.

Presentation of disaggregated data, accompanied by formal subgroup analyses where appropriate, would allow us to better understand the equity implications of interventions. It is important to note that subgroup analyses should be carefully planned and informed by well-formulated hypotheses: the higher the number of subgroup analyses, the higher the risk of spurious findings due to multiple statistical tests [47]. When planning a trial, investigators need to consider potential population differences in baseline risk of the condition or problem being studied and the possibility of differential effectiveness of the intervention and decide whether subgroup analyses are appropriate and can be accommodated in the sample-size calculation [48–50]. If additional subgroup hypotheses of interest are identified during the analyses, they should be clearly presented as exploratory and interpreted with caution [51–53]. Presentation of disaggregated results across relevant subgroups is important so that the data can be used in meta-synthesis. Reporting needs to improve to explicitly state and justify any pre-planned hypotheses and present results for all such analyses, describe relevant sample-size considerations, clearly identify exploratory analyses and their hypotheses, and report disaggregated data for relevant subgroups.

One mechanism to facilitate the availability of disaggregated data for meta-synthesis might be to share data in online repositories, which would allow meta-studies to be conducted which may have greater power to detect subgroup differences. In addition, trial registries could request details about planned subgroup analyses and the availability of equity variables. The CONSORT-Equity reporting guideline aims to improve the transparency and completeness of equity considerations in RCTs and CRTs by providing authors with a checklist for reporting. However, this reporting guideline is only one step towards more equity-relevant research which is needed to ensure that inequities are not perpetuated or worsened by programs and policies.

## Supplementary information

**Additional file 1: Appendix 1.** Search strategy.

**Additional file 2.**

## Data Availability

The dataset is available on Figshare or Open Science Framework (OSF).
