# Supplementary material for: Reporting of health equity considerations in cluster and individually randomized trials
Source: Trials. 2020 Apr 3;21:308. doi: 10.1186/s13063-020-4223-5 (PMC7118943; doi:10.1186/s13063-020-4223-5)
Supplement: Supplementary file 1 — Additional file 1: Appendix 1. Search strategy. [file 13063_2020_4223_MOESM1_ESM.docx]

Appendix 1 – Search Strategy

1. exp Gender Identity/ (16085)

2. (gender-based or gender-related or gender factors).tw. (5545)

3. ((sex or gender) adj3 (analysis or factor$ or inequit$ or disparit$ or inequalit$ or difference$ or interact$)).mp. [mp=title, abstract, original title, name of substance word, subject heading word, keyword heading word, protocol supplementary concept word, rare disease supplementary concept word, unique identifier] (283228)

4. exp sex factors/ (221712)

5. exp geriatrics/ (27357)

6. ((ethnic$ or race or racial or religio$ or cultur$ or minorit$ or refugee or indigenous or aboriginal or African american) adj3 (analysis or disparit$ or inequalit$ or inequit$ or difference$ or predict$ or interact$)).tw. (45723)

7. exp homosexuality/ (23786)

8. exp disabled persons/ (48889)

9. ((poverty or low-income or "lower income" or socioeconomic$ or socio-economic$ or social) adj3 (analysis or disadvantage$ or factor$ or inequalit$ or depriv$ or inequit$ or disparit$ or difference$ or predict$ or interact$)).tw. (60492)

10. exp Educational Status/ (42295)

11. exp Socioeconomic Factors/ (357728)

12. ((discriminat$ or social exclu$ or social inclu$) adj3 (religion or culture or race or racial or aboriginal or indigenous or ethnic$)).tw.( 1144)

13. ((urban or rural or inner-city or remote or slum) adj3 (analysis or inequit$ or disparit$ or inequalit$ or difference$ or predict$ or interact$)).tw. (4714)

14. ((resource-poor or ("low income" adj countr$) or ("middle income" adj countr$) or africa or developing countr$ or "south america" or china or asia or "latin america") adj3 (relevance or analysis or applicab$ or inequit$ or disparit$ or inequalit$ or difference$ or predict$ or interact$)).tw. (2899)

15. (inequalit$ or in-equalit$ or equit$ or inequit$ or in-equit$ or disparit$ or underserved or marginali$ed).tw. (75046)

16. exp indigenous populations/ (225586)

17. ((native* or Indian or aborigin*) adj3 (American* or Canadian* or Alaska*)).tw. (9720)

18. (first adj2 nation*).tw. (3585)

19. (aborigin$ or metis or inuit$ or eskimo$ or native or esquimaux or aleut or yuit or inughuit or unanga* or alutiiq or inup#ia* or kalaallit or Inuktitut or Nunavut or nunavik or cree or dene or haida or salish or Mohawk or ojibway or yupik or tribal or arctic).tw. (180806)

20. exp american native continental ancestry group/ or oceanic ancestry group/ (25767)

21. exp rural health/ (21983)

22. or/1-21 (1162565)

23. randomized controlled trial.pt. (405559)

24. (randomized or placebo).mp. (674403)

25. (cluster$ adj2 randomi$).tw. (5792)

26. or/23-25 (674953)

27. 22 and 26 (28463)

28. limit 27 to English (27659)

29. limit 28 to human (26420)

30. limit 29 to yr="2013 (**2549**)

limit 29 to yr="2014”(**2272**)

limit 29 to yr="2015” (**160)**
