# Supplementary material for: Reporting of health equity considerations in cluster and individually randomized trials
Source: Trials. 2020 Apr 3;21:308. doi: 10.1186/s13063-020-4223-5 (PMC7118943; doi:10.1186/s13063-020-4223-5)
Supplement: Supplementary file 2 — Additional file 2. [file 13063_2020_4223_MOESM2_ESM.docx]

1. Title: Is “equity” mentioned in title?
   1. Identify any specific equity-relevant terms in title (e.g., inequalities, unfair, disadvantage, vulnerable and copy full title OR if not relevant NR):
2. Abstract: Are any results reported across subgroups defined by PROGRESS?
   1. Does the “Plus” part of "PROGRESS-Plus" have any results reported across its defined subgroups?
3. Background: Are any anticipated differences in baseline risk or intervention acceptability, coverage, or effectiveness across subgroups defined by PROGRESS described?
   1. Are there additional differences in the aforementioned across subgroups as defined by the “Plus” part of “PROGRESS-Plus”?
4. Does the study provide an explicit objective pertaining to equity? (Example: Intervention aims to reduce avoidable, unfair, socially patterned differences in opportunities for health within the study population in terms of occurrence and/or uptake.)
5. Identify all PROGRESS Plus factors referred to in the study objective
   1. Place
   2. Race/ethnicity/language/culture
   3. Occupation
   4. Sex/gender
   5. Religion
   6. Education
   7. Socioeconomic status
   8. Social Capital
   9. Sexual orientation
   10. Plus 1 (age, disability, sexual preference)
   11. Plus 2 (feature of relationships)
   12. Plus 3 (time dependent relationships)
6. Is the study reported as situated in a resource constrained setting?
7. If Q6 is Yes, identify the study setting
   1. LMIC
   2. Public Hospital
   3. Conflict zone
   4. Other
8. Where were the participants recruited?
9. Identify the study population
   1. Students
   2. Workers
   3. Community members Patients
   4. Patients
   5. Members of a particular professional group
   6. Other
10. Identify the unit of randomization (i.e., what is the cluster?)
    1. Schools
    2. Workerplace
    3. Community or community organization
    4. Medical Practice
    5. Other
11. Are study eligibility criteria (ie. inclusion or exclusion criteria) defined across
    1. any PROGRESS characteristics?
    2. the additional “Plus” factors?
12. Type of equity-relevant trial classification:
    1. Targeted/focused
    2. Universal
    3. Both
13. Intervention type:
    1. structure-based
    2. Environment-based
    3. Behavior-based
    4. Individual therapeutic intervention
    5. Other
14. Are any baseline descriptive statistics for individuals or clusters defined by PROGRESS factors reported by trial arm, either in Table 1 or in text?:
    1. Place of residence
       1. Rural
       2. Urban
       3. Remote
       4. Not reported
       5. Other
    2. Race/ethnicity/culture/ language
    3. Occupation
    4. Gender/sex
    5. Religion
    6. Education
    7. Socioeconomic status
    8. Social capital
    9. Sexual orientation
    10. Plus 1: Any details about other personal characteristics attracting discrimination
    11. Plus 2: Any details about features of relationships affecting inequity
    12. Plus 3: Any details about time dependent circumstances that may affect inequity
15. What is the main PROGRESS factor of interest (reported in Q #14)
16. Does the trial present disaggregated baseline details across the main PROGRESS characteristic of interest?
17. Does the analysis adjust for any PROGRESS factors?
18. If Q17 is Yes, identify which PROGRESS factors are adjusted for in the analysis:
    1. Place
    2. Race/ethnicity/language/culture
    3. Occupation
    4. Sex/gender
    5. Religion
    6. Education
    7. Socioeconomic status
    8. Social Capital
    9. Sexual orientation
19. Identify the PRIMARY reported subgroup analysis (i.e., the subgroup analysis described in study objectives, or reported first, or described in title of study or mentioned in abstract) and identify the PROGRESS category using #'s 1-8 (where: 1-Place, 2-Race/ethnicity/language/culture, 3-occupation, 4-gender/sex, 5-religion, 6-education, 7-socioeconomic status, 8 social capital, 9 sexual orientation) OR NR if none
20. Is there any other reported subgroup analyses for PROGRESS? (e.g., intervention effects such as correlation analysis of effect on main outcomes and socioeconomic status)?
21. Consider the credibility criteria (Yusuf, 1991) for subgroup analyses as reported in the trial:
    1. Was the subgroup analysis pretested or planned a priori to the study commencement?
    2. Was there a hypothesis or rationale for the analysis provided?
    3. Was a statistical test for interaction performed between the subgroups?
    4. Were the overall treatment results emphasized more than the findings of the subgroup analysis
22. Is the sample size calculation reported as sufficiently powered to detect subgroup differences across the primary PROGRESS subgroup characteristic?
23. Are event rate differences (e.g., RR, OR, IRR, HR) or mean differences (intervention versus control) presented for each subgroup of the MAIN PROGRESS characteristics for each intervention?
    1. For the event rate difference for each subgroup, is difference between main PROGRESS characteristic levels expressed as a relative measure (e.g. relative risk, odds ratio, effect size relative to control group). This could be captured as the subgroup differences themselves, e.g., the Ratio of Odds.
    2. Is difference between main PROGRESS characteristic expressed as an absolute difference (e.g. risk differences between groups). This could be captured by means of an interaction term, or by means of separate estimates of treatment effect within each subgroup.
24. Are applicability/generalizability/external validity discussed across
    1. the MAIN PROGRESS characteristic of interest? (e.g., judgements about relevance to population, setting or time)
    2. the “Plus” characteristic of interest? (e.g., judgements about relevance to population, setting or time)
    3. in relation to the population in general?
25. Does the study report on any individual/community engagement processes?
26. Does the study report on any special or tailored recruitment procedures to increase enrolment of individuals who are members of disadvantaged populations?
27. Participant flow1: Does the study describe differential recruitment of individuals or clusters across PROGRESS (e.g., greater recruitment from higher income neighbourhoods than low-income neighbourhoods)
    1. In addition to PROGRESS, does the study describe differential recruitment of individuals or clusters across “Plus” characteristics?
28. Participant flow2: Does the study describe differential attrition across
    1. PROGRESS (e.g., more dropouts by single mothers than mothers in a couple; or more dropouts from low-income than higher-income participants)
    2. previously mentioned “Plus” characteristics?
29. Any identified concerns about adherence
    1. to the intervention by participants or compliance with providing the intervention by practitioners across PROGRESS (i.e., fidelity)?
    2. to the intervention by participants or compliance with providing the intervention by practitioners across “Plus” characteristics?
30. Any general comments regarding ethical concerns:
31. After reading this text, do you have any ethical concerns with regards to how the study was conducted? (e.g., Withholding vitamin supplements to undernourished children as a placebo for a controlled trial.)
